# Cycloart-24-ene-26-ol-3-one, a New Cycloartane Isolated from Leaves of *Aglaia exima* Triggers Tumour Necrosis Factor-Receptor 1-Mediated Caspase-Dependent Apoptosis in Colon Cancer Cell Line

**DOI:** 10.1371/journal.pone.0152652

**Published:** 2016-04-12

**Authors:** Kok Hoong Leong, Chung Yeng Looi, Xe-Min Loong, Foo Kit Cheah, Unang Supratman, Marc Litaudon, Mohd Rais Mustafa, Khalijah Awang

**Affiliations:** 1 Department of Pharmacy, Faculty of Medicine, University of Malaya, Kuala Lumpur, Malaysia; 2 Center for Natural Product and Drug Discovery (CENAR), Department of Chemistry, Faculty of Science, University of Malaya, Kuala Lumpur, Malaysia; 3 Department of Pharmacology, Faculty of Medicine, University of Malaya, Kuala Lumpur, Malaysia; 4 Department of Chemistry, Faculty of Science, University of Malaya, Kuala Lumpur, Malaysia; 5 Department of Chemistry, Faculty of Mathematics and Natural Sciences, Padjadjaran University, Jatinangor, Indonesia; 6 Centre de Recherche de Gif, Institut de Chimie des Substances Naturelles, CNRS 1, Avenue de la Terrasse, Gif-sur-Yvette Cedex, France; Rajiv Gandhi Centre for Biotechnology, INDIA

## Abstract

Plants in the Meliaceae family are known to possess interesting biological activities, such as antimalaral, antihypertensive and antitumour activities. Previously, our group reported the plant-derived compound cycloart-24-ene-26-ol-3-one isolated from the hexane extracts of *Aglaia exima* leaves, which shows cytotoxicity towards various cancer cell lines, in particular, colon cancer cell lines. In this report, we further demonstrate that cycloart-24-ene-26-ol-3-one, from here forth known as cycloartane, reduces the viability of the colon cancer cell lines HT-29 and CaCO-2 in a dose- and time-dependent manner. Further elucidation of the compound’s mechanism showed that it binds to tumour necrosis factor-receptor 1 (TNF-R1) leading to the initiation of caspase-8 and, through the activation of Bid, in the activation of caspase-9. This activity causes a reduction in mitochondrial membrane potential (MMP) and the release of cytochrome-C. The activation of caspase-8 and -9 both act to commit the cancer cells to apoptosis through downstream caspase-3/7 activation, PARP cleavage and the lack of NFkB translocation into the nucleus. A molecular docking study showed that the cycloartane binds to the receptor through a hydrophobic interaction with cysteine-96 and hydrogen bonds with lysine-75 and -132. The results show that further development of the cycloartane as an anti-cancer drug is worthwhile.

## Introduction

Cancer is a debilitating disease that affects a significant portion of the world’s population, and it is indeed a global health problem. Colorectal cancer remains one of the most prevalent cancers among patients in the United States, constituting 8% and 9% of all cancer cases for males and females, respectively [1]. Despite the recent advancements in cancer treatments, such as the development of targeted therapy [2], the relative survival rates for patients suffering from colorectal cancer have not improved significantly [3]. Moreover, chemotherapy using synthetic drugs often causes side effects, such as hair loss, bleeding, diarrhoea and myelotoxicity [4]. Researchers continue to search for new therapeutic agents that are more selective against cancer cells and that generate fewer side effects.

Plants remain one of the largest sources of natural products that are used to discover novel chemotherapeutic agents [5–6]. Notably, some novel compounds were discovered from plants that had unique mechanisms of action, greater potency or lower adverse effects than currently used drugs [7]. In collaboration with French institutions to search for novel medicinal drugs, we performed preliminary phytochemical profiling of the plant *Aglaia exima*. Aglaia is the largest genus of the Meliaceae family, and Aglaia trees commonly grow in tropical and subtropical forests in mainland China, Indo-Malaysian and the Pacific Islands. Aglaia tree parts (leaves, flowers and edible fruits) possess several medicinal properties, such as anti-inflammatory, anti-cancer, anti-diabetic properties. Six triterpenoids and two steroids were previously isolated from the leaves of *Aglaia exima* by our group. Previously, we showed that the new cycloartane exhibited the highest cytotoxic effect on the colon cancer cell line HT-29 of all the compounds isolated from *Aglaia exima* by our group, with an IC_50_ of 11.5 μM [8]. Interestingly, previous study reported cycloartane from *Aglaia* species displayed 10-fold selectivity towards colon cancer cell line HT-29 as compared to normal colon cell line CCD-112CoN [9].

The majority of current chemotherapy drugs trigger apoptosis to cause cancer cell death. Apoptosis is an active process of programmed cell death that occurs with specific morphological and biochemical changes in the cells [10]. These morphological changes include externalization of phosphatidylserine onto the cell surface, membrane blebbing, chromatin condensation and the formation of apoptotic bodies [11]. Progress in understanding the signalling of apoptosis has led to two major pathways of initiation being widely accepted, namely the extrinsic and intrinsic apoptosis pathways. The extrinsic pathway is triggered through death receptors present at the cell surface, whereas the intrinsic pathway is triggered by the release of proapototic factors, such as cytochrome c, from the cell’s mitochondria [12]. Tumour necrosis factor receptors, transmembrane proteins, are among the well-known external death receptors. These receptors include two types: tumour necrosis factor receptor-1 (TNF-R1) and -2 (TNF-R2). TNFR-1 is ubiquitously expressed in most cells, whereas TNFR-2 is mainly found in oligodendrocytes, astrocytes, T cells, myocytes, thymocytes, endothelial cells and mesenchymal stem cells [13]. The survival and death process is mainly regulated by TNF-R1, as this receptor contains an intracellular death domain that is not present in TNF-R2. Once activated, the death domain recruits other death signals, such as TRADD, FADD and pro-caspase-8, to form a death-inducing signalling-complex (DISC). The release of caspase 8 signals Bid to activate Bax, Bad, and cytochrome C in the cell’s mitochondria. Activation of TNR-R1 is believed to cause the metalloprotease TACE to release the extracellular component of the receptor as soluble TNF-R1 (sTNF-R1), which is a cytokine that is capable of activating other TNF-R1s to augment the death signals [14].

However, the main executioners of apoptotic pathways are proteases of the caspase family that proteolytically disintegrate the cells in the form of apoptotic bodies. This family of proteases is divided into executioner caspases, such as caspase 3 and 7, and initiator caspases, such as caspase 8 and 9. Initiator caspase-8 is known to be activated through the death receptors, whereas caspase-9 is activated by cytochrome c leakage from the mitochondria. These initiator caspases lead to downstream activation of caspase 3 and 7, committing the cell to apoptotic death. In contrast to necrosis, apoptosis is a non-inflammatory cell death pathway, which has the advantage of minimizing unwanted effects on neighbouring cells [15]. Therefore, scientists are constantly in search of small molecules that can trigger apoptosis in the treatment of cancer. In this study, we evaluated the anti-cancer potential of the cycloartane in human colon cancer cell line HT-29, focusing on the apoptotic mechanism underlying this activity.

## Materials and Methods

### Cell culture and cytotoxicity assay

Human colon cancer cell lines (HT-29 and CaCO-2) were obtained from the American Tissue Culture Collection (Rockville, MD). Cells were maintained in Dulbecco’s modified Eagle medium (DMEM) supplemented with 10% heat-inactivated foetal bovine serum (FBS), 2 mM L-glutamine, 50 μg/ml gentamycin and 2.5 μg/ml amphotericin B (Gibco, Carlsbad, CA) in a humidified incubator at 37°C with an atmosphere of 5% CO_2_.

The cytotoxicity of the cycloartane was evaluated against 2 colon cancer cell lines (HT-29 and CaCO-2). The cells were plated at a density of 5 x 10^3^ cells per well in 96-well plates and treated continuously with the compound (0.39–200 μM) or cisplatin (3.9–2000 μM) or 5-fluorouracil (200–8 x 10^−6^ mM) for 24, 48 and 72 hours. Cisplatin and 5-fluorouracil of more than 99.9% purity was obtained from Sigma-Aldrich. Control wells were treated with 0.05% v/v DMSO in the culture media. At the end of the treatment, the media was gently refreshed, and 20 μL of MTS reagent (CellTiter 96 AQ_ueous_® One Solution, Promega, Madison, WI) was added. The plates were incubated for 2 hours, and absorbance was read using a microplate reader (Infinite 200, Tecan, Männedorf, Switzerland) at 490 nm, with 690 nm as the background wavelength. The viable cell percentage was calculated with respect to control wells, and IC_50_ was determined using the dose-response curve fitted using Prism 5.02 software (GraphPad Software Inc., San Diego, CA). All experiments were performed in triplicate, and results are reported as arithmetic means ± SD.

### Apoptosis and cell cycle analyses

Apoptosis and cell cycle analyses were performed separately using commercial Annexin V: FITC Apoptosis Detection Kit I and CycleTest™ Plus DNA Reagent kit, respectively (BD Bioscience, San Jose, CA). Cells were seeded at 5 x 10^5^ cells per well in twelve-well plates. After overnight attachment, the cells were treated with the compound at 12.5, 25 and 50 μM for 24 hours for the apoptosis analysis and at 2.5 μM for 12, 24 and 48 hours for the cell cycle analysis. After treatment, the cells were harvested by gentle trypsinization and centrifuged at 1500 x g for 5 minutes. Cell staining was performed according to the manufacturer’s instructions. A DNA QC Particles kit was used to calibrate the FACSCanto II flow cytometer (BD Biosciences, San Jose, CA). Cell populations were subjected to cytometric analysis, and the quadrants were set according to the population of viable cells in the untreated samples. FacsDiva 5.0.3 software (BD Biosciences, San Jose, CA) was used to calculate the percent of cells in the respective quadrants for apoptosis analysis, and Mod Fit LT software (Verity Software House Inc., Topsham, ME) was used for the cell cycle analysis.

### Mitochondrial membrane potential ∆ѱm (MMP), cytochrome c release analysis and NFkB translocation

A Cellomics Multiparameter Cytotoxicity 3 Kit for MMP and cytochrome release and Nucleus Factor kappa B Activation Kit (ThermoFisher Scientific, Waltham, MA) were used as previously described [16]. Cells were plated at 1 x 10^4^ cells per well on 96-well plates overnight. Cells were washed and incubated with compound for 24 hours at various concentrations (3.125–50 μM). As for NFkB translocation activity, cells were treated at 12.5 μM of cycloartane alone or 10ng/ml TNF-α alone or 12.5 μM of cycloartane and 10ng/ml TNF-α in combination treatment. Then, the cells were fixed with 4% formaldehyde for 15 minutes and then permeabilized with 0.1% Triton X-100 in phosphate buffer saline (PBS). After fixation, the cells were incubated with MMP dye or blocked with 3% bovine serum albumin followed by NFkB primary rabbit antibody or cytochrome c primary mouse antibody and then goat anti-rabbit secondary antibody conjugated with DyLight^TM^ 488 or goat anti-mouse secondary antibody conjugated with DyLight^TM^ 649 for 1 hour each. The cells were rinsed three times with wash buffer II (1 X PBS with 1% Tween-20). The nuclei were stained with Hoechst 33258. Then, the stained cells were visualized, and the images were captured using Cellomics ArrayScan HCS reader ((ThermoFisher Scientific, Waltham, MA). The cell health profiling bioapplication module was used to quantify the fluorescence intensity of each dye.

### Caspase activity and inhibitor assays

Cells were plated in white 96-well plates at 1 x 10^4^ cells per well and were allowed to attach. Then, the cells were treated with the compound (50 μM) for different periods of time (1, 3, 6, 12, 18, 24 and 30 hours). Caspase activity was measured by adding 50 μl of Caspase-Glo® 3/7 (Z-DEVD-aminoluciferin), Caspase-Glo® 8 (Z-LETD-aminoluciferin) or Caspase-Glo® 9 (Z-LEHD-aminoluciferin) (Promega, Madison, WI) and then reading the luminescence using a microplate reader (Infinite 200, Tecan, Männedorf, Switzerland). The activities of the individual caspases were expressed as fold increases with respect to the untreated control. For the inhibitor assay, cells were pre-treated with 10 μM caspase 3 inhibitor (Z-DEVD-FMK), caspase 8 inhibitor (Z-IETD-FMK), caspase 9 inhibitor (Z-LEHD-FMK) or general caspase inhibitor (Z-VAD-FMK) for 30 minutes. Then the cells were incubated with the compound (50 μM) for 18 hours, and the caspase assays were performed as outlined above. After another 6 hours of incubation with the compound, cell viability was measured using the MTS reagent, as described in the cell culture and cytotoxicity assay section.

### Protein extraction, protein array and western blotting analyses

A total of 1 x 10^6^ cells were treated with the compound (50 μM) or TNFα (10ng/ml) as positive control for NFkB translocation, and changes in protein expression were monitored over time intervals of 6, 12, 18 and 24 hours. Cells were harvested, washed with ice-cold PBS and lysed in M-PER buffer containing Pierce 1x Halt protease inhibitor cocktail for total cellular extracts or NE-PER buffer for cellular nuclear extracts in NKkB experiments or Mem-PER Plus buffer for cellular membrane protein extracts in TNF-R1 experiments (ThermoFisher Scientific, Waltham, MA). For the protein array, the expression levels of 43 apoptosis-related proteins in the cell lysate were determined using the RayBio Human Apoptosis Array G1 kit, according to the supplier’s protocol. Fold changes between treated and untreated samples were analysed using the RayBio Antibody Array Analysis Tool (RayBiotech, Norcross, GA). For western blot, cell lysates (20 μg protein/well) were boiled for 5 minutes and then resolved on 12% sodium dodecyl sulphate–polyacrylamide gel electrophoresis (SDS–PAGE) and electrophoretically transferred to polyvinylidene difluoride (PVDF) membranes. The membranes were blocked with 5% non-fat milk in Tris-buffered saline containing 0.5% Tween 20 (TBST) for 1 hour and incubated with primary antibody overnight at 4°C. The kits used were the apoptosis antibody sampler kit (#9915), pro-apoptosis Bcl-2 family antibody sampler kit (#9942), and death receptor sampler kit (#8356), and the antibodies used were the nuclear factor-kappa B p65 (#8242), beta-actin (#8457) and lamin B2 (#13823) antibodies (Cell Signaling, Dancers, DA). Subsequently, the membranes were washed with TBST buffer and incubated with the appropriate horseradish peroxidase-conjugated secondary antibody (goat anti-rabbit IgG) (#7074). Membranes were developed using an enhanced chemiluminescence kit (Super Signal West Dura, ThermoFisher Scientific, Waltham, MA).

### Computational molecular docking and statistical analyses

The X-ray crystal structure of tumour necrosis factor receptor 1 (TNF-R1) was retrieved from the Protein Data Bank (PDB entry 1NCF) and prepared using the CHARMM force field. Docking was performed using the c-DOCKER module of Discovery Studio 4.1 software (Acceryls, San Diego, CA) to simulate the interactions between the compound and TNF-R1. The binding interaction energy was calculated using the *in-situ* minimization tool to incorporate flexible docking at the docked site, following a previously described protocol [17]. Statistical analyses were performed using the student’s t-test or ANOVA with Bonferroni post-test in Prism 5.02 software to determine significant differences between the experimental and control groups (GraphPad Software Inc., La Jolla, CA), and statistical significance was defined as p ≤ 0.05 or p ≤ 0.01.

## Results

### Cytotoxic effect of cycloartane on HT-29 and CaCO-2 colon cancer cell-lines

**Fig 1:**
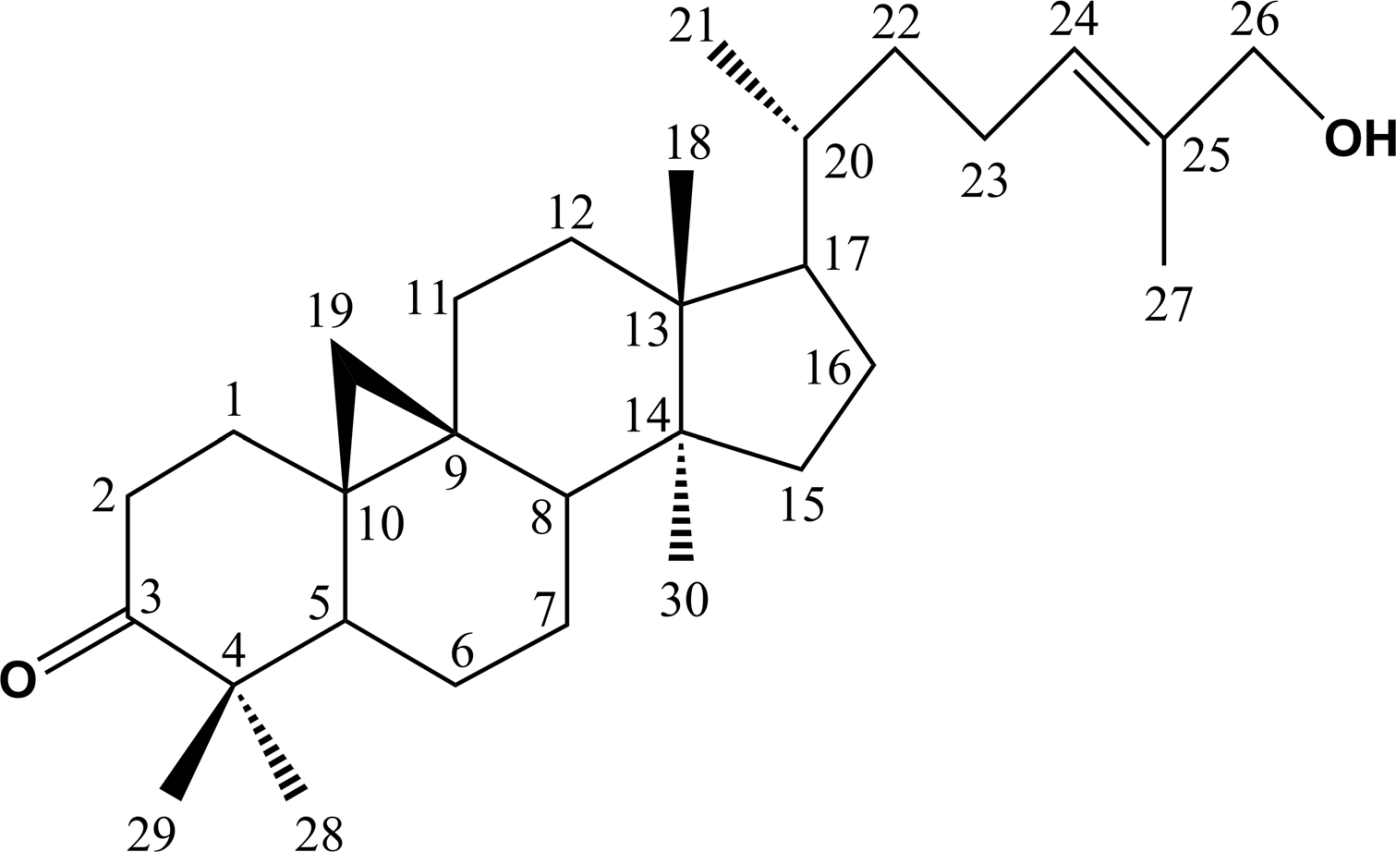
Chemical structure of cycloart-24-ene-26-ol-3-one. The compound has a cyclopentano per hydro phenanthrene scaffold with a cyclopropane ring between C-9 and -10. The side chain attached to C-17 has a hydroxyl group substituent at C-26.

**Fig 2:**
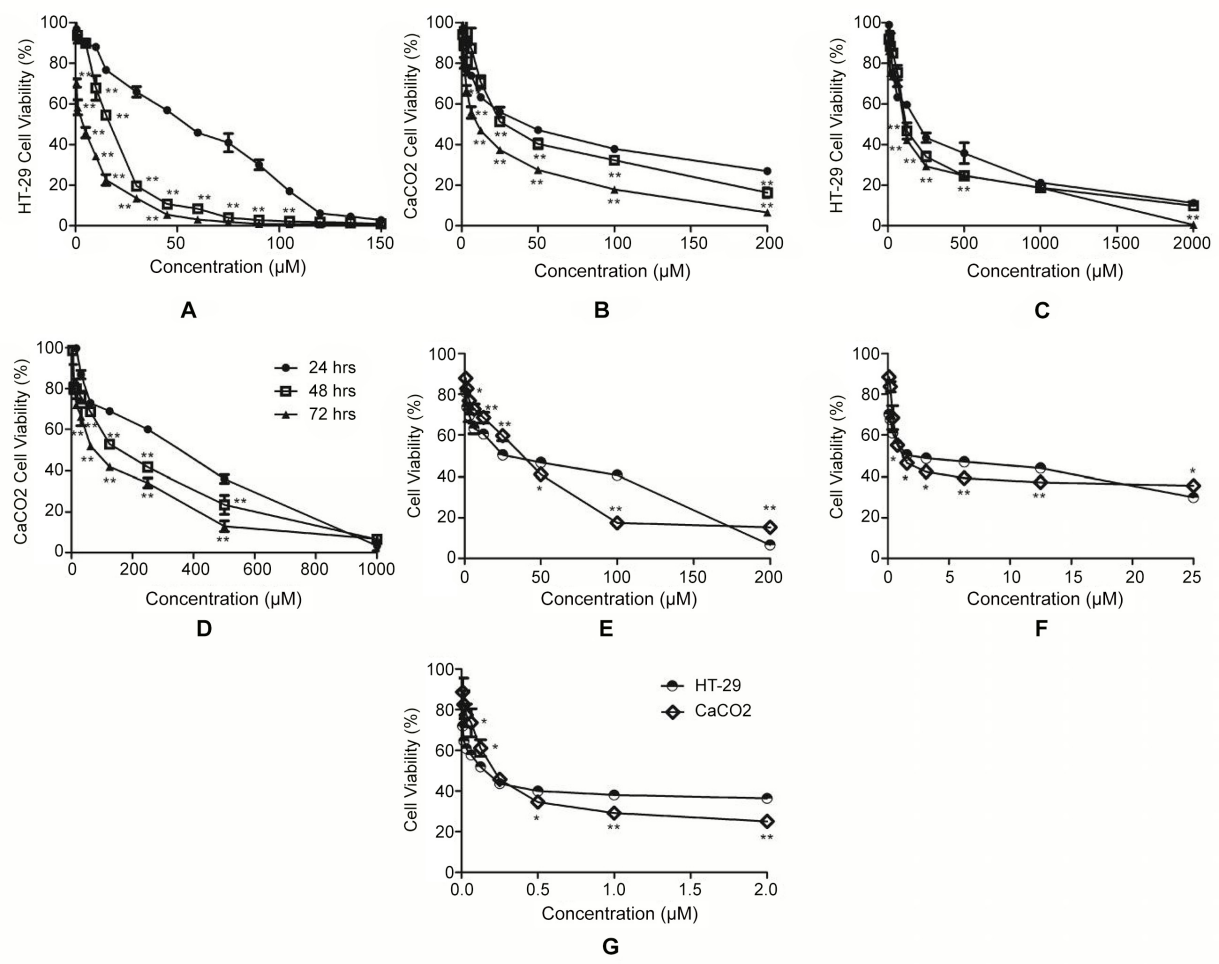
Dose- and time-response curves. For cycloartane (0.39–200 μM) on HT-29 (A) and CaCO-2 (B) cell lines, cisplatin (3.9–2000 μM) on HT-29 (C) and CaCO-2 (D) cell lines at 24, 48 and 72 hours, 5-fluorouracil (200–8 x 10^−6^ mM) on HT-29 and CaCO-2 cell lines at 24 (E), 48 (F) and 72 (G) hours. Significant differences are indicated: *p < 0.05; **p < 0.01.

The chemical structure of the cycloartane is shown in Fig 1. Both, HT-29 and CaCO-2 cells were treated with different dosages 0.39–200 μM of the compound for 24, 48 and 72 hours. Cell viability was determined by MTS assay. As shown in Fig 2, the compound reduced cell viability in a dose- and time-dependent manner. In addition, the IC_50_ values of the cycloartane were 3–30 fold lower than that of the standard chemodrug cisplatin (3.9–2000 μM) at each treatment time-point, 42–862 fold lower compared to 5-fluorouracil at 24 and 48 hour time-points but 16–27 fold more potent after 72 hours treatment (Table 1). The IC_50_ values obtained were used as a guide for the subsequent experiments.

**Table 1:** The IC_50_ of the cycloartane, cisplatin and 5-fluorouracil on HT-29 and CaCO-2 cells for 24-, 48- and 72-hour treatments (n = 3).

Hours	IC_50_ of the cycloartane		IC_50_ of cisplatin		IC_50_ of 5-fluorouracil	
	(Mean ± SD μM)		(Mean ± SD μM)		(Mean ± SD)	
	HT-29	CaCO-2	HT-29	CaCO-2	HT-29	CaCO-2
24	51.6 ± 3.5	41.6 ± 2.6	181.1 ± 6.1	120.9 ± 7.0	25.98 ± 3.11 mM	35.83 ± 1.79 mM
48	11.5 ± 0.5	22.8 ± 1.5	104.5 ± 1.2	93.5 ± 3	1.43 ± 0.11 mM	0.95 ± 0.10 mM
72	2.4 ± 0.1	5.6 ± 0.1	75.7 ± 4.7	62.3 ± 1.2	0.15 ± 0.01 μM	0.21 ± 0.02 μM

### Cycloartane induces cell cycle arrest and apoptosis in HT-29 cells

**Fig 3:**
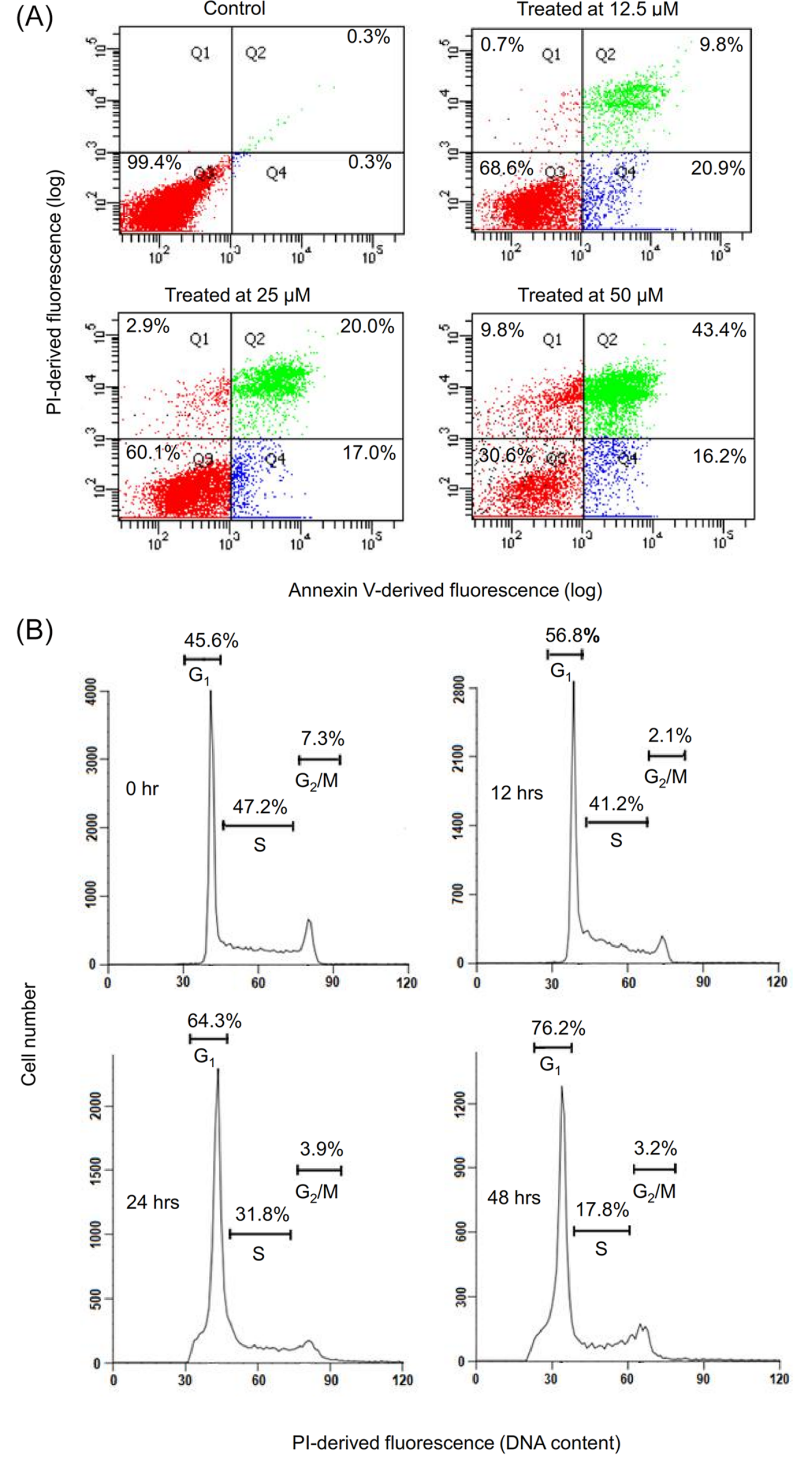
Effect of the cycloartane on HT-29 cell death (apoptosis and necrosis) and cell cycle progression. (A) Percentage of cells showing phosphatidylserine translocation, as measured by cell-surface annexin V binding and free PI: cells negative for annexin V and positive for PI are necrotic (Q1); cells positive for both annexin V and PI are in late apoptosis (Q2); cells negative for both annexin V and PI (Q3) are viable cells; and cells positive for annexin V and negative for PI are in early apoptosis (Q4). Cells were incubated for 24 hours with 0.1% v/v DMSO_4_ (vehicle control) or the cycloartane at concentrations of 12.5, 25, or 50 μM, as indicated. (B) Flow cytometry histograms showing the distribution of cells in different phases of the cell cycle (G_1_, S and G_2_/M) at the beginning of the experiment (0 hr) and at 12, 24 and 48 hours of treatment with 2.5 μM of the cycloartane. The result is representative of one of three replicates that had essentially similar results.

To evaluate the mode of cell death caused by the cycloartane, HT-29 colon cancer cells were treated with the compound for 24 hours. Next, we performed flow cytometry analysis of cells that were double-stained with annexin-V and propidium iodide (PI). The control cells were treated with vehicle (0.1% v/v DMSO_4_) and had 99.4% cell viability, with 0.6% of cells in apoptosis. Treatment with the compound at increasing concentrations, from 12.5 μM to 50 μM, caused reduced cell viability from 68.6% to 30.6% and a concomitant increase in the percentage of apoptotic cells from 30.7% to 59.6% and the percentage of necrotic cells from 0.7% to 9.8% (Fig 3A).

Next, we investigated the cell cycle profile of the cycloartane-treated HT-29 cells. As shown in Fig 3B, the flow cytometry analysis showed a time-dependent increase of cells in G_1_ phase, with 45.6%, 56.8%, 64.3% and 76.2% of cells being in G_1_ phase after 0, 12, 24 and 48 hours of treatment, respectively.

### Cycloartane induces activation of caspases 3/7, 8 and 9

**Fig 4:**
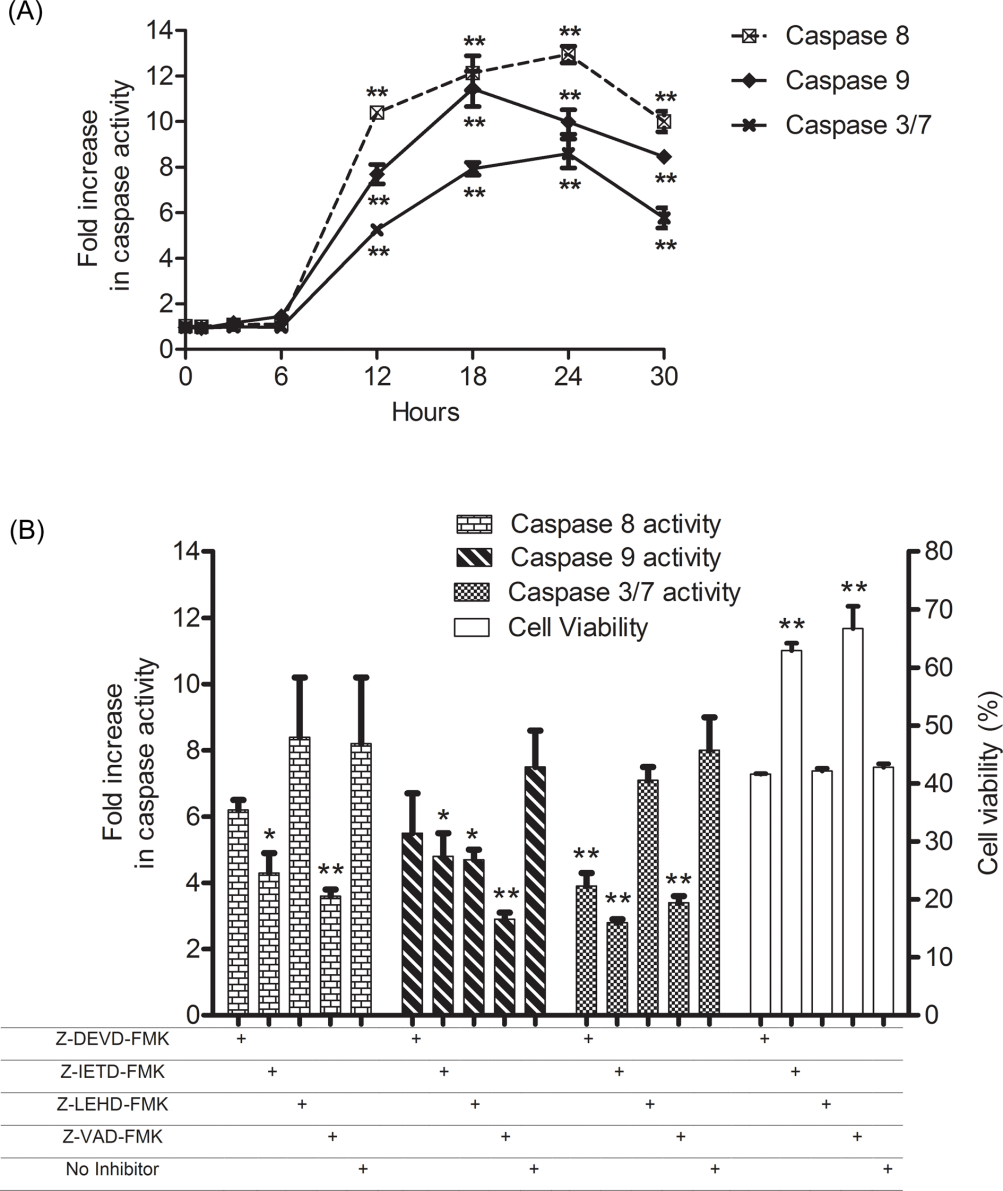
Fold changes in caspase activities and cell viability of treated HT-29 cells with respect to untreated control. (A) Time course (0–30 hours) fold increase in caspase 3/7, 8 and 9 activities of HT-29 cells post-treatment with the cycloartane (50 μM). (B) Effects of caspase inhibitors on treated HT-29 cells. Changes in caspase 3/7, 8 and 9 activities, and cell viability of cells pre-treated with caspase 3 inhibitor (Z-DEVD-FMK), caspase 8 inhibitor (Z-IETD-FMK), caspase 9 inhibitor (Z-LEHD-FMK) or general caspase inhibitor (Z-VAD-FMK), followed by incubation with the cycloartane (50 μM). At 18 hours of treatment, caspase activities were determined, and at 24 hours of treatment cell viability was measured. All results are three independent determinations with fold increases and percent cell viabilities calculated based on the untreated control. Symbols indicate significantly higher compared to 0 hour or no inhibitor pre-treatment: *p < 0.05; **p < 0.01.

Caspases play a central role in apoptosis-induction, and as shown in Fig 4A, HT-29 cells treated with the compound showed a sharp increase in the activities of caspases 3/7, 8 and 9 from 6 to 12 hours. Their activities peaked at 18 hours for caspase 9 and 24 hours for caspase 3/7 and 8, which was followed by a decreasing trend. Pre-treatment with either caspase 8 (Z-IED-FMK) or a pan-caspase inhibitor (Z-VAD-FMK) followed by the cycloartane resulted in higher cell viability than in cells without pre-treatment. However, inhibitors for caspase 3 (Z-DEVD-FMK) and 9 (Z-LEHD-FMK) did not rescue the cells from the cycloartane-induced apoptosis (Fig 4B).

### Effects of cycloartane on apoptosis signalling proteins

**Fig 5:**
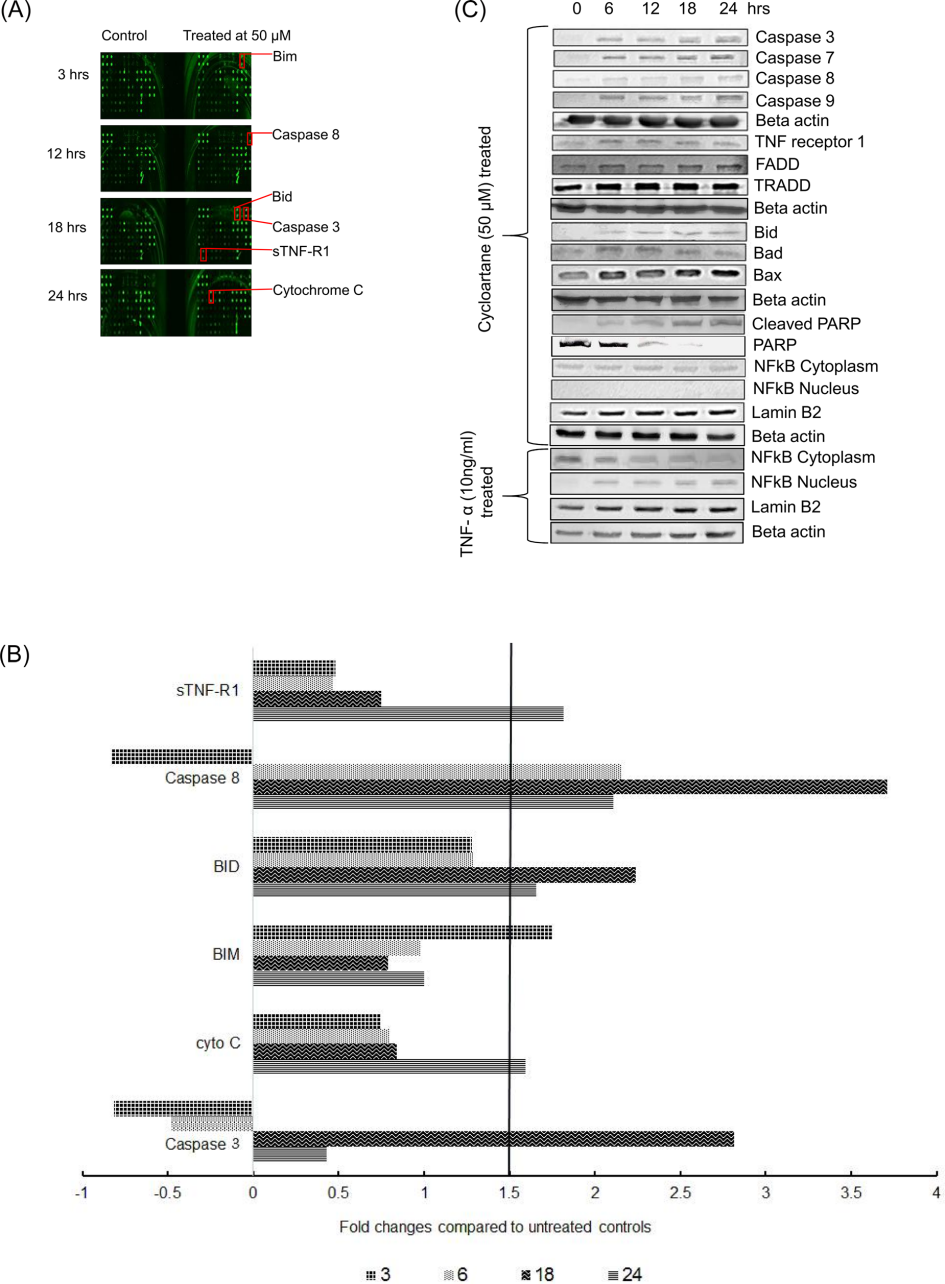
Expression of apoptosis signalling proteins in cycloartane-treated HT-29 cells over various time intervals (6, 12, 18 and 24 hours). (A) Upregulated proteins detected in human apoptosis antibody array. (B) Fold changes of apoptosis signalling molecules in comparison to control with a cut off limit of 1.5 fold. (C) Western blot analysis of apoptotic signalling proteins in cycloartane treated HT-29 cells and TNF-α-treated as positive control for NFkB translocation over various time intervals (6, 12, 18 and 24 hours).

Next, we collected protein lysates at 6, 12, 18, and 24 hours after treatment with the cycloartane and loaded them onto the RayBio Human Apoptosis Array to detect the levels of 43 apoptosis-inducing proteins. The greatest increase in protein expression was seen for caspase 8, which increased from 2 to 4 fold at the 12- and 18-hour time-points. Caspase 3 and Bid only increased after 18 hours of the compound treatment (Fig 5A and 5B).

Whole cell lysates of cells treated with the cycloartane were collected at different time-points and subjected to Western blotting analysis. Consistent with the protein array results (Fig 5A and 5B), time-dependent increases in Bid and cleaved caspase 3 and 8 proteins (Fig 5C) were observed. Also, TNF-R1, FADD, and TRADD protein expressions were up-regulated, indicating that the compound induced the extrinsic apoptosis signalling pathway through the TNF-R1 receptor (Fig 5C); sTNF-R1 was also shown to be up-regulated by the protein array results (Fig 5A and 5B). The mitochondrial-related pro-apoptotic proteins Bax and Bad also increased significantly from 6 to 24 hours. In addition, no translocation of NFkB from the cytoplasm to the nucleus was observed over time and the level of cleaved PARP increased over time. Cells treated with 10ng/ml TNF-α served as a positive control for the NFkB translocation experiments (Fig 5C).

### Cycloartane reduces mitochondrial membrane potential (MMP), increases cytochrome c release and NFkB translocation events

**Fig 6:**
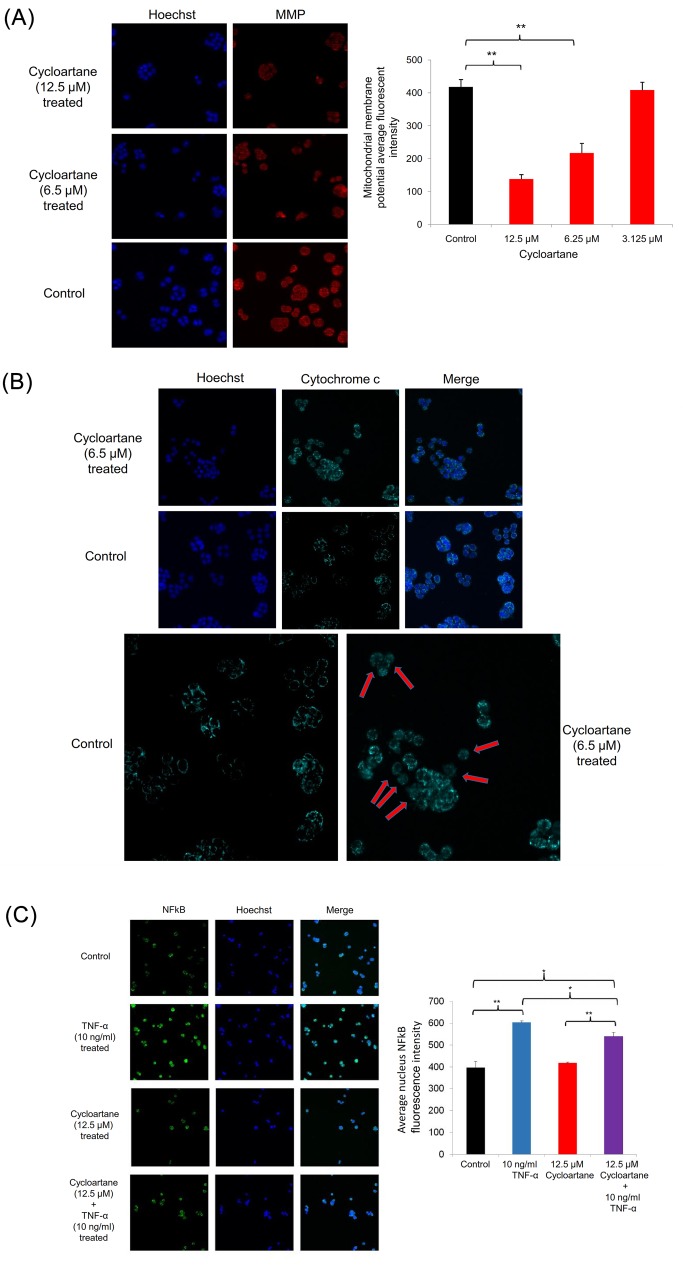
High content cell screening analysis. (A) Images and bar chart of mitochondrial membrane potential (MMP) decrease. (B) Cytochrome c localization (red arrows) in control cells or release from cycloartane-treated HT29 colon cancer cells. (C) NFkB fluorescence intensity in the nucleus region are similar between control and cycloartane-treated cells, reduce intensity in combination treatment of cycloartane and TNF-α or increase intensity in TNF-α alone. Images were captured at 100X magnification and significant differences are indicated: *p < 0.05; **p < 0.01.

To examine whether the compound causes mitochondrial damage and cytochrome c release, we treated HT-29 cells with the compound for 24 hours and then performed a high content cell screening analysis. As shown in Fig 6A, the cycloartane at 6.25 μM and 12.5 μM decreased (p<0.01) MMP. In addition, we observed cytochrome c release into the cytosol of HT-29 cells treated with 6.25 μM of the compound in comparison with the untreated control (Fig 6B). In examining whether the compound compete with the natural ligand, TNF-α, the downstream NFkB translocation into the nucleus was measured. In Fig 6C, treatment of TNF-α (10ng/ml) resulted in NFkB fluorescence intensity increase (p<0.01) in the nucleus region. However, treatment with cycloartane (12.5 μM) did not significantly (p>0.05) increase NFkB fluorescence intensity compared to control. Combination treatment of cycloartane (12.5 μM) and TNF-α (10ng/ml) resulted in a significant (p<0.05) decrease in NFkB fluorescence intensity compared to TNF-α treatment alone.

### Study of cycloartane docking to TNF-R1

**Fig 7:**
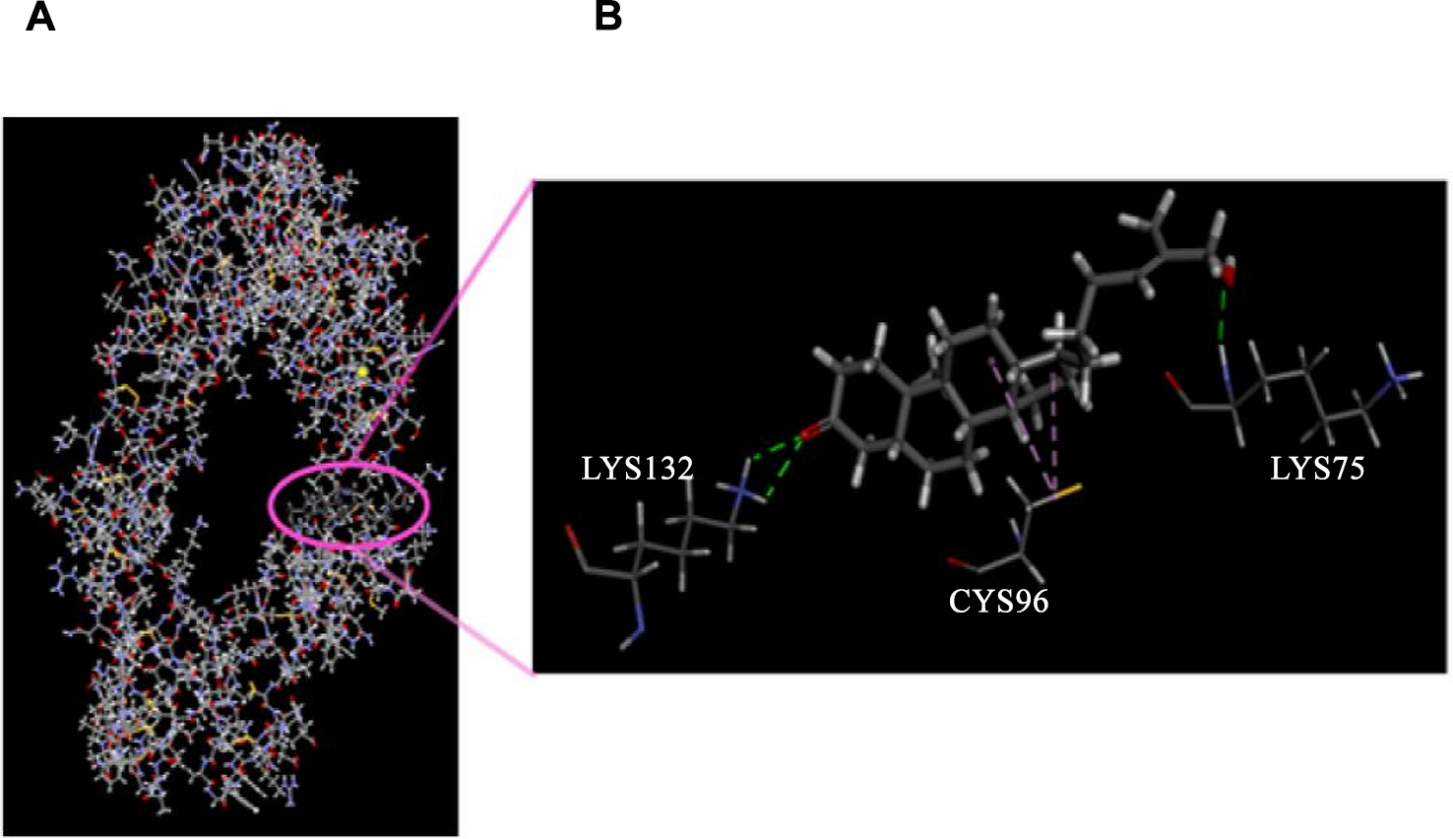
Molecular docking of the cycloartane (circle) onto TNF-R1. Magnification showing hydrogen bonding (green dashed lines) between hydroxyl and carbonyl groups of the compound with lysine-75 and -132 and hydrophobic interactions (purple dashed lines) between the cyclohexane and cyclopentane of the compound with cysteine-96 of the receptor.

Computational molecular docking illustrated the binding of the compound to TNF-R1 (Fig 7) and revealed a total binding energy of -67.032 kcal. The binding site for the cycloartane on TNF-R1 differs from the active site for the natural ligand, TNFα. The compound binds to the extracellular domain near the cell membrane. Hydrophobic interaction was observed between one of the cyclohexane and cyclopentane groups of the compound and cysteine-96, and hydrogen bonds formed between the hydroxyl at C-26 and carbonyl groups at C-3 of the compound with lysine-75 and -132 of the receptor, respectively.

## Discussion

Our previous cytotoxic screening of a panel of cancer cell lines showed that the cycloartane is most cytotoxic towards the colon cancer cell line HT-29 [8]. In this study, we observed similar cytotoxic effects in another human colon cancer cell line, CaCO-2. The results of the MTS assays show that treatment with the compound causes a dose- and time-dependent reduction in cell viability in both HT-29 and CaCO-2 cells. As the IC_50_ of the compound was lowest for the HT-29 cell line, further experiments were performed in that cell line.

Phosphatidylserine externalization on the cell surface is one of the hallmarks of apoptosis [15]. Flow cytometry studies showed a concentration-dependent increase in annexin-V FITC cell populations compared to PI cell populations, indicating that this compound induces apoptosis rather than necrosis. In addition, the accumulation of cells in G_1_ stage over time was detected by flow cytometry after the cycloartane treatment. Normal cell growth and differentiation require proper regulation of the cell cycle. Deregulated cell cycle control due to mutation, deletion and transcriptional repression of genes, such as FBXW7, pRB, and p53, has been shown to contribute to colorectal cancer progression [18–20]. Thus, inhibiting cell cycle progression could stop uncontrolled proliferation of colon cancer cells and cause them to enter apoptosis.

Another hallmark of apoptosis is the activation of caspases, and there are two established pathways based on the initiator caspases: the death receptor pathway, which involves caspase-8, and the mitochondrial pathway, which involves caspase-9 [16]. Initiation of either or both caspases activates the executioner caspase (caspase 3/7), which ultimately leads to well-regulated cell demise. Incubation with the compound causes a time-dependent activation of caspase 3/7 from the 6^th^ hour onwards. The increase in caspase-8 activation was concomitant with the increase of caspase-9 activation, suggesting the involvement of both death receptor and mitochondrial pathways in the induction of apoptosis by the cycloartane. However, it is unclear which caspase is the primary initiator based on the trend of caspase activation. To determine this, we performed a caspase inhibitor experiment. The caspase 8 inhibitor (Z-IETD-FMK) was able to suppress caspase 9 and 3/7 activities leading to an increase in viable cells. Therefore, caspase 8 is the primary initiator, whereas caspase 9 is a secondary mediator that amplifies the apoptotic signal.

Further investigation of the apoptotic pathway by protein array analysis demonstrated the release of soluble tumour necrosis factor-receptor 1 (sTNF-R1) from whole cell extracts. The activation of TNF-R1 is believed to cause the metalloprotease TACE to release the extracellular component of the receptor as soluble TNF-R1 (sTNF-R1) [14], leading us to believe that the TNF-R1 was triggered in the upstream event. Western blot analysis of cellular membrane extract of the treated cells confirmed the upregulation of TNF-R1. TNF-R1 belongs to the death receptor family, and the cytoplasmic tail of TNF-R1 contains a death domain (DD), which is essential for the induction of apoptosis [21, 22]. Studies revealed that TNF-R1 activates the apoptotic program by sequential recruitment of the adaptor protein TRADD, the TRADD-interacting adaptor protein FADD and the FADD-like ICE protein (FLICE, also called pro-caspase-8) to form the death-inducing signalling complex (DISC) [23, 24]. Activation of the TNF-R1 receptor by drugs has been shown to augment treatment efficiencies in both drug-resistant cell lines and primary carcinoma cells [25].

**Fig 8:**
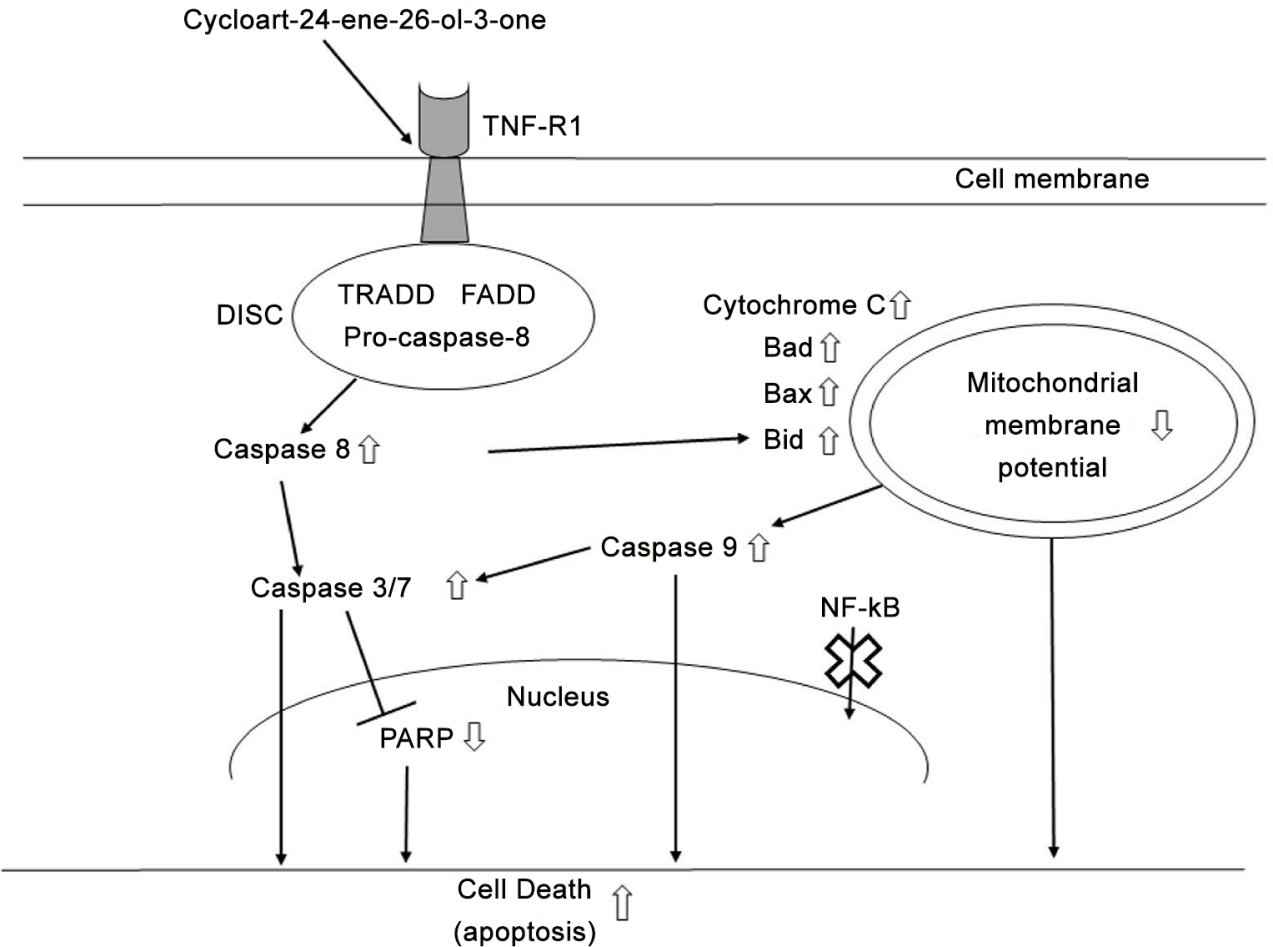
The apoptotic effects of the cycloartane in HT-29 cells. The diagram illustrates the mechanism of apoptotic death when the cycloartane binds to tumour necrosis factor receptor 1 (TNF-R1). This action causes recruitment of TRADD, FADD and pro-caspase-8 to form the death-inducing signalling-complex (DISC). The release of caspase 8 signals Bid to activate Bax, Bad, cytochrome C and caspase 9, leading to mitochondrial membrane potential decrease. Both caspase 8 and 9 activate caspase 3/7, which inhibits PARP and causes DNA degradation, resulting in apoptotic death. No translocation of NKĸB from the cytoplasm to the nucleus was observed. Thick upward and downward arrows indicate increases or decreases, respectively, of expression levels or activities. Thick cross indicates that the signalling protein (NFĸB) did not migrate to the nucleus.

TNF-R1 activation further induces Bid, which activates Bad and Bax, causing mitochondrial membrane potential to decrease, cytochrome c to be released and caspase 9 to be activated. The role of Bid and Bad in transducing apoptotic signals from death receptors to the intrinsic mitochondria pathway involves amplification of the apoptotic signals [26]. Bad and Bax belong to the Bcl-2 family members, which regulate cell death and survival. For example, the upregulation of Bax has been shown to induce apoptosis by reducing mitochondrial permeability to release cytochrome c [27]. The consequence of cytochrome c release and caspase 9 activation is the activation of downstream caspases 3 and 7, which causes cleavage of PARP and leads to apoptotic death without the translocation of NFkB to the nucleus. Further fluorescence labelling of NFkB showed the compound did not significantly (p>0.05) increase NFkB translocation into the nucleus compared to control. More importantly, combination treatment of cells with the compound and TNF-α could significantly (p<0.05) reduce NFkB translocation as compared to TNF-α alone. The result showed that the cyclocartane was able to compete with TNF-α for the receptor in reducing downstream NFkB translocation. Additionally, the molecular docking study showed that the cycloartane binds to TNF-R1 via hydrophobic interactions and hydrogen bonds, confirming the affinity of the compound for TNF-R1. In short, the cycloartane induced apoptosis in HT-29 colon cancer cells that mimics the TNF-induced apoptosis possibly by binding through TNFR1, which needs further investigation. This action further activated the intrinsic pathway, and the two pathways worked in combination to commit the cell to apoptosis, as summarized in Fig 8. Initial research into using the natural ligand of TNF-R1, TNF-α, to activate the receptor for anticancer treatment was halted due to the adverse effects of inflammation and septic shock [28]. Although TNF-α was shown to be effective in inducing caspase-dependent apoptosis, it also induced the NFkB-mediated inflammatory response. Furthermore, it was found that prolonged activation of NFkB lead to drug resistance and, increases angiogenesis and metastasis [29]. This outcome has brought about challenges in using TNF-α for anticancer treatment [30, 31]. Reports on plant-derived compounds that bind to TNF-R1 without activating NFkB are rare [32, 33]. The ability of the cycloartane to activate TNF-R1 in causing caspase-dependent apoptosis in our results follow the similar apoptosis pathway as in TNF-α. The exception for this compound compared to TNF-α is that no significant NFkB activation was observed in treated HT-29 adenocarcinoma cells. This suggest the anti-cancer potential of this compound possibly without the adverse effects seen in TNF-α, which warrants further investigation of this compound.

## Supporting Information

S1 Fig The molecular structures of compounds previously published. (PDF)
