# Supplementary material for: Cycloart-24-ene-26-ol-3-one, a New Cycloartane Isolated from Leaves of Aglaia exima Triggers Tumour Necrosis Factor-Receptor 1-Mediated Caspase-Dependent Apoptosis in Colon Cancer Cell Line
Source: PLoS One. 2016 Apr 12;11(4):e0152652. doi: 10.1371/journal.pone.0152652 (PMC4829234; doi:10.1371/journal.pone.0152652)
Supplement: S1 Fig — (PDF) [file pone.0152652.s001.pdf]

Fitoterapia 83 (2012) 1391–1395

Contents lists available at SciVerse ScienceDirect

Fitoterapia

journal homepage: www.elsevier.com/locate/fitote

Triterpenes and steroids from the leaves of *Aglaia exima* (Meliaceae)☆

Khalijah Awang [a],*, Xe-Min Loong [a], Kok Hoong Leong [b], Unang Supratman [c], Marc Litaudon [d], Mat Ropi Mukhtar [a], Khalit Mohamad [b]

[a] *Department of Chemistry, University of Malaya, 50603 Kuala Lumpur, Malaysia*
[b] *Department of Pharmacy, University of Malaya, 50603 Kuala Lumpur, Malaysia*
[c] *Department of Chemistry, Faculty of Mathematics and Natural Sciences, Padjadjaran University, 45363 Jatinangor, Indonesia*
[d] *Centre de Recherche de Gif, Institut de Chimie des Substances Naturelles, CNRS, 1, Avenue de la Terrasse, 91198 Gif-sur-Yvette Cedex, France*

ARTICLE INFO

Available online 23 October 2012

Keywords:
Aglaia exima
Meliaceae
Cycloartane
Cytotoxicity
Steroids
24(*E*)-cycloart-24-ene-26-ol-3-one

ABSTRACT

A study on the leaves of *Aglaia exima* led to the isolation of one new and seven known compounds: six triterpenoids and two steroids. Their structures were elucidated and analyzed mainly by using spectroscopic methods; 1D and 2D NMR, mass spectrometry, UV spectrometry and X-ray. All the triterpenoids and steroids were measured *in vitro* for their cytotoxic activities against eight cancer cell lines; lung (A549), prostate (DU-145), skin (SK-MEL-5), pancreatic (BxPC-3), liver (Hep G2), colon (HT-29), breast (MCF-7) and (MDA-MB-231). The new cycloartane triterpenoid, 24(*E*)-cycloart-24-ene-26-ol-3-one **1**, showed potent cytotoxic activity against colon (HT-29) cancer cell line (IC_{50} 11.5 μM).

© 2012 Elsevier B.V. All rights reserved.

1. Introduction

The genus *Aglaia* is the largest genus of the family Meliaceae which has a total of 105 species and is widely distributed in subtropical and tropical forest of southern mainland China, Indo-Malaysian region and the Pacific Island [1]. Meliaceae is a family known for the presence of triterpenes which possess interesting biological activities such as hypoglycemia, anticancer, anti-inflammatory, antifeedant, insecticides and antitumor activities [2,3].

In the framework of our ongoing French-Malaysia Collaborative program [4–6], we have initiated the phytochemical investigation on the leaves of *Aglaia exima*. In this study, eight compounds, including five cycloartane-type triterpenoids (**1**–**5**); 24(*E*)-cycloart-24-ene-26-ol-3-one **1**, cycloart-24-ene-3β,26-diol **2** [7,8], schizandronic acid **3** [9], 24(*E*)-3β-hydroxycycloart-24-ene-26-al **4** [10], vaticinone **5** [11], one dammarane-type triterpenoids; cabraleahydroxylactone **6** [12,13], and two steroids (**7**–**8**); β-sitosterol **7** [14–16] and stigmast-5-ene-28-one **8** [17], were isolated from the leaves of *A. exima* (Fig. 1). We herein report the isolation and structure elucidation of the new cycloartane; 24(*E*)-cycloart-24-ene-26-ol-3-one **1**, together with the cytotoxicities of compounds **1**–**7** against eight cancer cell lines: lung (A549), prostate (DU-145), skin (SK-MEL-5), pancreatic (BxPC-3), liver (Hep G2), colon (HT-29) and breast (MDA-MB-231).

2. Experimental

2.1. General

The specific rotations were determined on Jasco P-1020 Polarimeter. UV spectra were measured by using Shimazu UV-160A ultraviolet–visible spectrometer with methanol. IR spectra were recorded by Perkin Elmer 1600 Series FT-IR. ^{1}H, ^{13}C,

☆ This article is dedicated to Prof. Dr. Atta-ur-Rahman who is a great inspiration to all Natural Products scientists.

* Corresponding author.
E-mail address: khalijah@um.edu.my (K. Awang).

0367-326X/$ – see front matter © 2012 Elsevier B.V. All rights reserved.
http://dx.doi.org/10.1016/j.fitote.2012.10.004

DEPT, HSQC and HMBC NMR spectra were measured on JEOL JNM-LA 400 FT-NMR and JEOL ECA 400. Mass spectra were obtained by Shimadzu LCMS-IT-TOF Liquid Chromatography Mass Spectrometer and Shimadzu GCMS-QP2010. Solvents were distilled prior to use, and spectroscopic grade solvents were employed. Column chromatography (CC) was carried out on Merck silica gel 60 (70–230 mesh and 230–400 mesh) and TLC on silica gel Merck 60 GF_{254}. Spots on the plates were detected under UV light and visualized by spraying with vanillin reagent followed by heating.

2.2. Plant material

The leaves of *A. exima* was collected from H.S. Kg. Kepayang, Pahang, Malaysia on November 1997 and identified by Mr. Teo Leong Eng (University of Malaya). Voucher specimen (KL 4762) has been deposited at the Herbarium of the Department of Chemistry, University of Malaya, Kuala Lumpur, Malaysia.

2.3. Plant extraction

Dried ground leaves (1 kg) of *A. exima* were extracted exhaustively with hexane at room temperature for 4 days and then filtered. The solution was decanted and then evaporated to give a residue of 25 g hexane extracts. 15 g of the hexane crude was subjected to column chromatography over silica gel using a gradient mixture of hexane and ethyl acetate as eluent. A total of 123 fractions were obtained and Fr. 54 gave crystals which is compound **7** (34.2 mg). Further purification of Fr. 67 (hexane: ethyl acetate 86:14, 0.72 g) by CC with silica gel gave **5** (6.5 mg, hexane: ethyl acetate 92:8), **1** (19.1 mg, hexane: ethyl acetate 90:10), **4** (25.6 mg, hexane: ethyl acetate 89:11) and **8** (5.1 mg, hexane: ethyl acetate 88:12). Fr. 94 to Fr. 100 (hexane: ethyl acetate 60:40 → 20:80, 1.5398 g) were combined and further purified by CC on silica gel to furnish **3** (16.3 mg, hexane: ethyl acetate 78:22) and **6** (2.9 mg, hexane: ethyl acetate 90:10). Of 123 fractions, Fr. 92 was collected as crystals which were then recrystallized by ethyl acetate to give a colorless crystal **2** (93.6 mg).

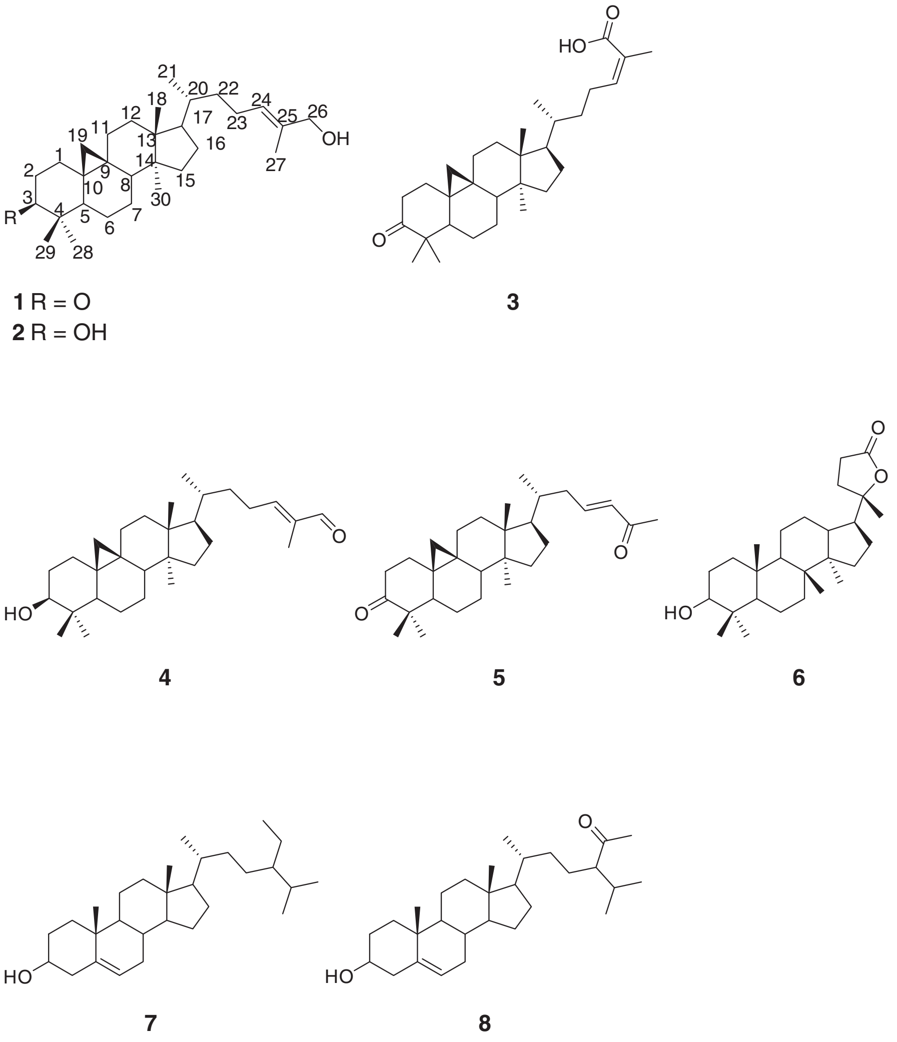

Fig. 1. The molecular structure of compounds **1**–**8**.

2.4. Spectroscopic data of compounds

2.4.1. Compound **1**

Colorless amorphous solid, $[\mu]_D^{27.7}$ +29.7° (c 0.00209, CH_2Cl_2); IR (KBr) υ_{max}: 3445 (O–H stretch), 2942 (C–H stretch), 1705 (C=O ketone stretch); UV (MeOH): 237 nm; ESI-TOF: *m/z* 463.3813 $[M+Na]^+$ calculated *m/z* 463.3678, ^{1}H- and ^{13}C-NMR data: see Table 1.

2.4.2. Compound **8**

Colorless crystal; $[\propto]_D^{27.4}$ −75.0° (c 0.00008, CH_2Cl_2); IR (KBr) υ_{max}: 3419 (O–H stretch), 2934 (C–H stretch), 2852 (C–H stretch), 1713 cm^{-1} (C=O ketone stretch); UV (MeOH): 208 nm; EI-MS (*m/z* rel int): 428 *m/z*; ^{1}H- and ^{13}C-NMR data: see Table 2.

Table 1
^{1}H and ^{13}C NMR of compounds **1** and **8** in $CDCl_3$.

Position	δH (ppm)	δC (ppm)	δH (ppm)	δC (ppm)
1	1.48 (*m*)<br>1.78 (*m*)	33.4	1.06 (*m*)<br>1.79 (*m*)	37.3
2	2.30 (*ddd*, J_1 = 4.1 Hz, J_2 = 8.2 Hz, J_3 = 14.4 Hz)<br>2.70 (*dt*, J_1 = 6.4 Hz, J_2 = 14.2 Hz)	37.4	1.80 (*m*)	31.7
3	–	216.7	3.50 (*m*)	71.9
4	–	50.2	2.21 (*m*)	42.4
5	1.52 (*m*)	48.4	–	140.8
6	1.50 (*m*)	21.5	5.32 (*d*, *J* = 6.9 Hz)	121.8
7	1.22 (*m*)<br>1.84 (*m*)	28.1	1.44 (*m*)<br>1.92 (*m*)	32.0
8	1.64 (*m*)	47.8	1.44 (*m*)	32.0
9	–	21.1	0.89 (*m*)	50.1
10	–	25.9	–	36.5
11	1.10 (*m*)<br>1.98 (*m*)	26.7	1.40 (*m*)<br>1.44 (*m*)	21.1
12	1.60 (*m*)	32.8	1.10 (*m*)<br>1.96 (*m*)	39.8
13	–	45.3	–	42.4
14	–	48.7	0.96 (*m*)	55.9
15	1.02 (*m*)	35.9	0.98 (*m*)<br>1.52 (*m*)	24.3
16	1.86 (*m*)<br>2.02 (*m*)	24.5	1.14 (*m*)<br>1.74 (*m*)	28.2
17	–	52.2	1.04 (*m*)	55.8
18	1.00 (*s*)	18.1	0.70 (*s*)	11.9
19	0.58 (*d*, *J* = 4.26 Hz)<br>0.79 (*d*, *J* = 4.26 Hz)	29.5	1.00 (*s*)	20.1
20	1.02 (*m*)	35.9	1.32 (*m*)	35.9
21	0.91 (*d*, *J* = 6.1 Hz)	18.2	0.91 (*d*, *J* = 2.0 Hz)	18.5
22	1.24 (*m*)	35.5	0.88 (*m*)<br>1.20 (*m*)	33.9
23	1.08 (*m*)<br>1.30 (*m*)	25.8	1.32 (*m*)<br>1.54 (*m*)	25.4
24	5.36 (*t*, J_1 = 7.1 Hz)	127.0	2.11 (*d*, *J* = 6.9 Hz)	60.6
25	–	134.3	1.78 (*m*)	30.2
26	3.94 (*s*)	69.0	0.87 (*d*, *J* = 2.2 Hz)	21.3
27	1.68 (*s*)	13.6	0.89 (*d*, *J* = 2.2 Hz)	20.1
28	1.05 (*s*)	22.2	–	213.5
29	1.10 (*s*)	20.7	2.09 (*s*)	30.0
30	0.91 (*s*)	19.3		

2.5. In vitro assay for cytotoxic activity

2.5.1. Cell lines

The hexane extract of the leaves from *A. exima* was investigated for cytotoxic activity against eight cancer cell lines; lung (A549), prostate (DU-145), skin (SK-MEL-5), pancreatic (BxPC-3), liver (Hep G2), colon (HT-29), breast (MCF-7) and (MDA-MB-231). These cancer cell lines were chosen from the National Cancer Institute (NCI) list of 60 cancer cell lines for drug screening and drug treatment conditions were done according to the NCI recommendations (Boyd, 1995). The human cancer cell lines were obtained from the American Type Culture Collection (ATCC) (Manassas, VA, USA). Dulbecco's modified Eagle's medium (DMEM), 100 mM non-essential amino acids, phosphate buffer solution (pH 7.2), 50 μg/ml gentamicin and 2.5 μg/ml amphotericin B were purchased from Invitrogen Corporation (Carlsbad, CA, USA). 200 mM L-glutamine, fetal bovine serum, 0.25% trypsin-EDTA, dimethyl sulfoxide (DMSO), cisplatin and vinblastine sulfate were purchased from Sigma-Aldrich (St. Louis, MO, USA). MTS [3-(4,5-dimethylthiazol-2-yl)-5-(3-carboxymethoxyphenyl)-2-(4-sulphophenyl)-2H-tetrazolium, inner salt] assay kit (CellTiter 96® AQ_{ueous} One Solution) was obtained from Promega (Madison, WI, USA).

2.5.2. Cytotoxic assay

Cell lines were cultured in DMEM media supplemented with 2 mM L-glutamine, 10% fetal bovine serum, 50 μg/ml gentamicin and 2.5 μg/ml amphotericin B, maintained in a 37 °C humid atmosphere of 5% CO_2 cell incubator. Samples and drug standards (cisplatin and vinblastine sulfate) were dissolved in DMSO and immediately diluted with DMEM media to yield a final DMSO concentration of less than 0.5% v/v.

Cells were plated into 96-well microplates at 5000–10,000 cells per well and maintained in the cell incubator for 24 h. Then, 100 μL of samples was introduced in triplicates to a final concentration of 15–200 μM, with the exception of sample **1** that was further diluted down to 4 μM in BxPC-3 and HT-29 cell lines. Drug standards were also introduced to a final concentration of 0.03–2000 μM (cisplatin) and 0.002–100 μM (vinblastine sulfate). Cells were further incubated for 48 h and then, cell viability was determined according to the manufacturer protocol of a commercial MTS assay kit (CellTiter 96 AQ_{ueous}® One Solution, Promega). Culture media were carefully refreshed with 100 μL of DMEM media, followed by 20 μL per well of MTS reagent. Microplates were returned to the incubator for 1 to 2 h and absorbance of the formazan product was read on a microplate reader at 490 nm with 690 nm as the background wavelength (Infinite 200, Tecan, Männedorf, Switzerland). IC_{50} of samples and drug standards were determined using dose–response curves in Prism 5.02 software (GraphPad Software Inc., La Jolla, CA, USA).

3. Results and discussion

Compound **1**, was obtained as a colorless amorphous solid, $[\propto]_D^{27.7}$ +29.7° (c 0.00209, CH_2Cl_2). The ESI-TOF spectrum showed an $[M+Na]^+$ pseudomolecular ion peak at m/z 463.3813 (calcd 463.3678) which corresponded to the molecular formula of $C_{30}H_{48}O_2$. The IR spectrum showed absorption peaks at 3445, 2942, and 1705 cm^{-1} suggesting the

Table 2
IC_{50} values of compounds/standard drugs for eight cancer cell lines [a].

Name of compounds	A549	DU-145	SK-MEL-5	BxPC-3	Hep G2	HT-29	MCF-7	MDA-MB-231
24(*E*)-cycloart-24-ene-26-ol-3-one **1**	–	–	96.6	–	–	11.5	86.2	–
Cycloart-24-ene-3β,26-diol **2**	172.4	–	157.8	–	75.1	99.3	127.7	195.2
Schizandronic acid **3**	–	–	–	–	–	–	–	–
24(E)-3β-hydroxycycloart-24-Ene-26-al **4**	–	–	117.8	–	–	–	–	94.4
Vaticinone **5**	–	–	105.7	–	–	96.8	–	–
Cabraleahydroxylactone **6**	–	–	–	–	–	–	–	–
β-Sitosterol **7**	–	–	–	–	–	–	–	–
Stigmast-5-ene-28-one **8**	–	–	–	–	–	–	–	–
Cisplatin	36.2	12.5	68.9	22.1	15.2	70.2	90.1	306.7
Vinblastine	29.0	4.8	1.7	2.0	0.4	1.0	28.1	35.3

[a] Results are expressed as IC_{50} values in μM. Blank indicates IC_{50} more than 200 μM.

presence of hydroxyl, alkyl and carbonyl groups respectively. The cycloartane nature of **1** was deduced by the appearance of a pair of much shielded doublets at δ 0.58 and 0.79 (J = 4.4 Hz). The olefinic proton (H-24) resonated as a broad triplet at δ 5.36 (J = 7.1 Hz). In addition, the oxymethylene protons which were attached to C-26 appeared as a broad singlet at δ 3.94. The ^{13}C NMR and DEPT spectra showed peaks corresponding to thirty carbons; six methyl, twelve methylenes, five methines, and seven quaternary carbons. The peak at δ 216.7 is assignable to the ketonic carbonyl, C-3. The HMBC spectrum confirmed the position of the C-3 carbonyl by showing correlations of H_2-2, H_3-28 and H_3-29 with the C-3 carbonyl (Fig. 2). In addition, the signals of the double bond carbons (C-24, C-25) appeared at δ 127.0 and δ 134.3 respectively. The methylene carbon C-26 of the side chain resonated downfield at δ 69.0 since it is attached to a hydroxyl group. The HMBC spectrum showed correlations of H-24 with C-26 and C-27, H-26 with C-24, C-25 and C-27 thus confirming the position of the double bond at C-24 and C-25 respectively. Furthermore, the location of the hydroxyl group on C-26 was also established by the HMBC correlations of H_2-26 with C-24, 25 and C-27. Thorough analysis of the DEPT, COSY, HSQC and HMBC spectra allowed the complete assignment of all protons and carbons (Table 1). The NOESY experiment showed a correlation between H-24 and H_2-26 thus suggesting that the C-24–C-25 double bond assumes an *E*-configuration. Therefore, compound **1** was elucidated as 24(*E*)-cycloart-24-ene-26-ol-3-one.

Compounds **2**–**7** were isolated by repeated column chromatography and characterized by comparison of their NMR data with literature values, known compounds **2**–**7** were

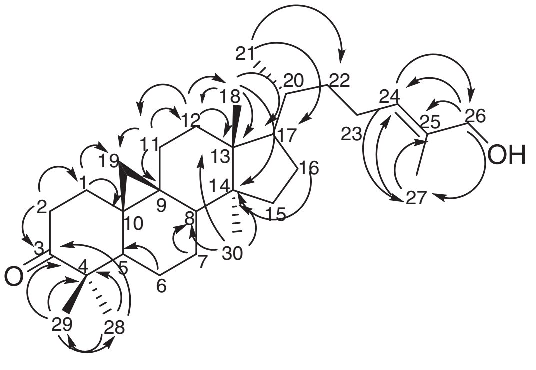

Fig. 2. Selected HMBC correlations of compound **1**.

identified as cycloart-24-ene-3β,26-diol **2** [7,8], schizandronic acid **3** [9], 24(E)-3β-hydroxycycloart-24-ene-26-al **4** [10], vaticinone **5** [11], cabraleahydroxylactone **6** [112,13] and β-sitosterol **7** [14–16]. Compound **8** is a new natural product. It was previously synthesized by Nobuo et al. [17]. The complete proton and carbon assignments of **8** are listed in Table 1.

Colon (HT-29) cancer cell line was found to be very susceptible towards 24(E)-cycloart-24-ene-26-ol-3-one **1** with IC_{50} values of 11.48 μM. Meanwhile, 24(E)-cycloart-24-ene-26-ol-3-one **1** revealed moderate to skin (SK-MEL-5) and breast (MCF-7). Cycloart-24-ene-3β, 26-diol **2** shows a moderate effect against liver (Hep G2) and colon (HT-29), weak against lung (A549), skin (SK-MEL-5), breast (MCF-7) and (MDA-MB-231). Vaticinone **5** revealed moderate inhibitory effect towards colon (HT-29) and weak towards skin (SK-MEL-5). At last, 24(E)-3β-hydroxycycloart-24-ene-26-al **4** has a moderate effect against breast (MDA-MB-231) and weak against skin (SK-MEL-5). Schizandronic acid **3**, cabraleahydroxylactone **6**, β-sitosterol **7** and stigmast-5-ene-28-one **8** exhibited no significant inhibitory effects with IC_{50} values over 200 μM.

Acknowledgment

This work was supported by grant from the Institute of Research Management and Monitoring (IPPP) (PS344/2009C), Fundamental Research Grant Scheme (FRGS) (FP006/2011A) and Research University Grant (RP001/2012A). We thank D. M. Nor (University of Malaya) for the collection of plant material and Mr. Teo Leong Eng for the botanical identification. This work was carried out within the framework of an official agreement between the University of Malaya (Malaysia) and CNRS (France).

References

[1] Su BN, Chai H, Mi Q, Riswan S, Kardono LBS, Afriastini JJ, et al. Bioorg Med Chem 2006;14:960-72.
[2] Janaki S, Vijayasekaran V, Viswanathan S, Balakrishna K. J Ethnopharmacol 1999;67:45-51.
[3] (a) Taylor DAH. In: Herz W, Grisebach H, Kirby GW, editors. In progress in the chemistry of organic natural products, vol. 45. New York: Springer; 1984.
(b) Mulholland DA, Parel B, Coombes PH. Curr Org Chem 1984;4: 1011-54.
(c) Awang K, Lim CS, Mohamad K, Morita H, Hirasawa Y, Takeya K, Thoison O. Bioorg Med Chem 1984;15:5997-6002.
Nagoor NH, Muttiah NSJ, Chong SL, Lionel LA, Mohamad K, Awang K. PLoS One 1984;6(8):e23661.

[4] Harneti D, Tjokronegoro R, Safari A, Supratman U, Loong XM, Mukhtar MR, et al. Phytochem Lett 2012;5:496-9.
[5] Mohamad K, Hirasawa Y, Litaudon M, Awang K, Hadi AHA, Takeya K, et al. Bioorg Med Chem 2009;17:727-30.
[6] Mohamad K, Sevenet T, Dumontet V, Pais M, Van Tri M, Hadi H, et al. Phytochemistry 1999;51:1031-7.
[7] Anjaneyulu V, Prasad KH, Ravi K, Connolly JD. Phytochemistry 1985;24:2359-67.
[8] Awang K, Loong XM, Mohamad K, Chong SL, Ng SW. Acta Crystallogr E 2010;66:2142.
[9] Sy LK, Saunders RMK, Brown GD. Phytochemistry 1997;44:1099-108.
[10] Januario AH, Silva MFDGFD, Viera PC, Fernandes JB. Phytochemistry 1992;28:1471-7.
[11] Zhang HJ, Tan GT, Hoang VD, Hung NV, Cuong NM, Soejarto DD, et al. J Nat Prod 2003;66:263-8.
[12] Phongmaykin J, Kumamoto T, Ishikawa T, Suttisri R, Saifah E. Arch Pharmacal Res 2008;31:21-7.
[13] Loong XM, Mohamad K, Awang K, Hadi AHA, Ng SW. Acta Crystallogr Sec E 2010;66:2541.
[14] Usuki S, Ariga T, Somsankar D, Kasama T, Morikawa K, Nonaka S, et al. J Lipid Res 2008;49:2188-96.
[15] Kang WY, Li GH, Hao XJ. Acta Bot Sin 2003;45:1003-7.
[16] Boonyaratavej S, Petsom A. J Sci Soc Thai 1991;17:61-9.
[17] Nobuo I, Masuo M, Hiroshi O, Yukiko C. J Chem Soc [Sec D]: Chem Commun 1971;22:1498.
